# Targeting TRAP1 as a downstream effector of BRAF cytoprotective pathway: A novel strategy for human BRAF-driven colorectal carcinoma

**DOI:** 10.18632/oncotarget.4263

**Published:** 2015-06-13

**Authors:** Valentina Condelli, Francesca Maddalena, Lorenza Sisinni, Giacomo Lettini, Danilo Swann Matassa, Annamaria Piscazzi, Giuseppe Palladino, Maria Rosaria Amoroso, Franca Esposito, Matteo Landriscina

**Affiliations:** ^1^ Laboratory of Pre-Clinical and Translational Research, IRCCS, Referral Cancer Center of Basilicata, Rionero in Vulture, PZ, Italy; ^2^ Department of Molecular Medicine and Medical Biotechnology, University of Naples Federico II, Naples, Italy; ^3^ Medical Oncology Unit, Department of Medical and Surgical Sciences, University of Foggia, Foggia, Italy

**Keywords:** BRAF, TRAP1, apoptosis, colon cancer, drug resistance

## Abstract

The HSP90 chaperone TRAP1 is translational regulator of BRAF synthesis/ubiquitination, since BRAF down-regulation, ERK signaling inhibition and delay of cell cycle progression occur upon TRAP1 silencing/inhibition. Since TRAP1 is upregulated in human colorectal carcinomas (CRCs) and involved in protection from apoptosis and as human BRAF-driven CRCs are poorly responsive to anticancer therapies, the relationship between TRAP1 regulation of mitochondrial apoptotic pathway and BRAF antiapoptotic signaling has been further evaluated. This study reports that BRAF cytoprotective signaling involves TRAP1-dependent inhibition of the mitochondrial apoptotic pathway. It is worth noting that BRAF and TRAP1 interact and that the activation of BRAF signaling results in enhanced TRAP1 serine-phosphorylation, a condition associated with resistance to apoptosis. Consistently, a BRAF dominant-negative mutant prevents TRAP1 serine phosphorylation and restores drug sensitivity in BRAFV600E CRC drug-resistant cells with high TRAP1 levels. In addition, TRAP1 targeting by the mitochondria-directed HSP90 chaperones inhibitor gamitrinib induces apoptosis and inhibits colony formation in BRAF-driven CRC cells. Thus, TRAP1 is a downstream effector of BRAF cytoprotective pathway in mitochondria and TRAP1 targeting may represent a novel strategy to improve the activity of proapoptotic agents in BRAF-driven CRC cells.

## INTRODUCTION

BRAF is one of the top12 mutant genes in human malignancies, with the substitution at position 600 from a valine to a glutamic acid (BRAF-V600E) the most common [1, 2]. Human BRAF-driven tumors, mostly melanomas, and thyroid and colorectal carcinomas, are biologically and clinically aggressive malignancies, frequently resistant to conventional anticancer therapies [3, 4]. Indeed, the oncogenic activation of BRAF drives the inappropriate activation of ERK signaling and the deregulation of cell proliferation [5], and is responsible for the inhibition of the mitochondrial apoptotic pathway [6–8], the latter being consistent with the apoptosis-resistant phenotype of BRAF-driven cancer cells. In this perspective, BRAF translocation to mitochondria represents a prerequisite for enabling resistance to apoptosis and this results in inhibition of cytochrome c release and inactivation of the caspase cascade [7], although the molecular mechanisms of BRAF antiapoptotic responses in mitochondria are not fully elucidated. From a clinical perspective, BRAF-mutated colorectal carcinomas (CRCs) are frequently addicted to this mitochondrial survival pathway, resistant to apoptosis and poorly responsive to standard chemotherapeutics and EGFR monoclonals [9–11]. Thus, the molecular characterization of BRAF-dependent antiapoptotic mechanisms is the prerequisite for targeting the BRAF survival pathway, thus representing a major clinical need, based on the lack of appropriate and effective treatments for these tumors [11].

Recent evidence by our group suggests that TRAP1 is responsible for the translational regulation of BRAF synthesis/ubiquitination in CRC cells [12]. Indeed, TRAP1 is a molecular chaperone, a member of the HSP90 chaperone family, involved in the maintenance of mitochondrial integrity and regulation of mitochondrial transition pore (MTP) [13], and upregulated in several human malignancies including CRCs [13–16]. Several lines of evidence suggest that TRAP1 is responsible for dual control on mitochondrial apoptotic pathway: i) folding/stability regulation on cyclophillin D and, likely, other client proteins critical for MTP opening within mitochondria [13, 17, 18], and ii) quality control regulation on specific client proteins in the endoplasmic reticulum (ER), most of which are extremely important regulators of mitochondrial apoptosis [15, 18–20]. In this context, our group has previously demonstrated that TRAP1 i) interacts with the proteasome regulatory protein particle TBP7 in the ER, ii) is involved in extra-mitochondrial quality control of nuclear-encoded proteins through co-translational regulation of their ubiquitination/synthesis, and iii) induces parallel activation of a cytoprotective UPR response and consequent protection from apoptosis [15, 19]. In this context, BRAF synthesis/ubiquitination is tightly regulated by ER-associated TRAP1, as an additional and non redundant mechanism respect to HSP90 control of BRAF stability [12, 21]. Intriguingly, while BRAF synthesis is induced in a TRAP1-rich background, its ubiquitination is enhanced upon disruption of TRAP1 network, in correlation to decreased protein levels. It is worth noting that this regulation is conserved in human malignancies, since the two proteins are significantly co-expressed in human CRCs, thus representing a potential therapeutic window for tumor-selective targeting of BRAF-driven colorectal malignancies [12].

Based on this well-characterized TRAP1 cytoprotective network and the knowledge that the RAS-RAF-ERK axis drives extracellular survival stimuli to mitochondria [4], we evaluated the relationship between TRAP1 regulation of MTP and BRAF signaling in mitochondria, with this study reporting that TRAP1 is a downstream effector of the BRAF cytoprotective pathway.

## RESULTS

### BRAF induces a cell phenotype resistant to apoptosis by modulating the mitochondrial apoptotic pathway

**Figure 1:**
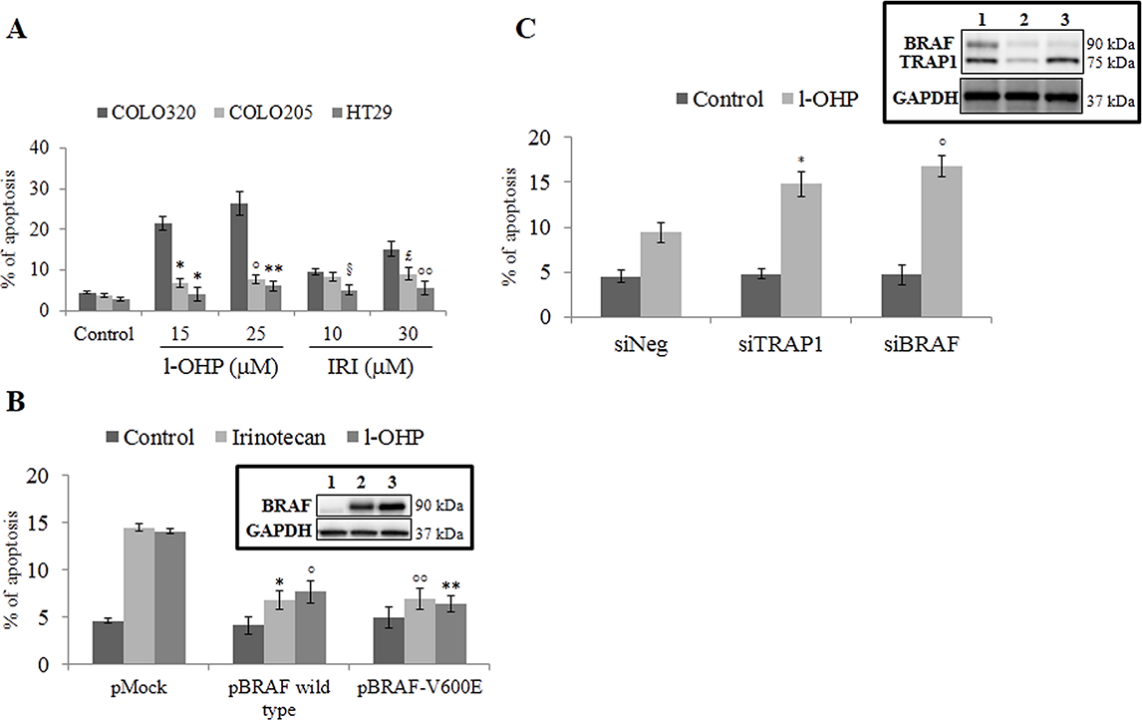
Activation of BRAF signaling protects from apoptosis **A.** Apoptotic cell death in BRAF wild type COLO320 cells and BRAF-V600E HT29 and COLO205 cells exposed to the indicated concentrations of oxaliplatin (l-OHP) and irinotecan (IRI) for 24 h. Statistical significance respect to COLO320 cells treated with the same agent: *p = 0.0003; °*p* = 0.0005; **p = 0.0004; ^§^*p* = 0.005; ^£^*p* = 0.01; °°*p* = 0.003. **B.** Apoptotic cell death in BRAF wild type COLO320 cells transfected with BRAF wild type or BRAF-V600E constructs and treated with 10 μM l-OHP or IRI for 24 h. Statistical significance respect to pMock cells treated with l-OHP: *p = 0.006; °*p* = 0.001. **Insert**: Immunoblot analysis of BRAF expression in COLO320 cells transfected with pMock (1), pBRAF wild type (2) or pBRAF-V600E (3) constructs. **C.** Apoptotic cell death in TRAP1- or BRAF-transiently silenced BRAF-V600E HT29 cells treated with 10 μM l-OHP for 24 h. Statistical significance respect to siNeg cells treated with the same agent: *p = 0.0006; °°*p* = 0.0009; °*p* = 0.002; **p = 0.0007;. **Insert**: Immunoblot analysis of BRAF and TRAP1 expression in HT29 cells transfected with Negative (1), TRAP1 (2) and BRAF (3) siRNAs.

**Figure 2:**
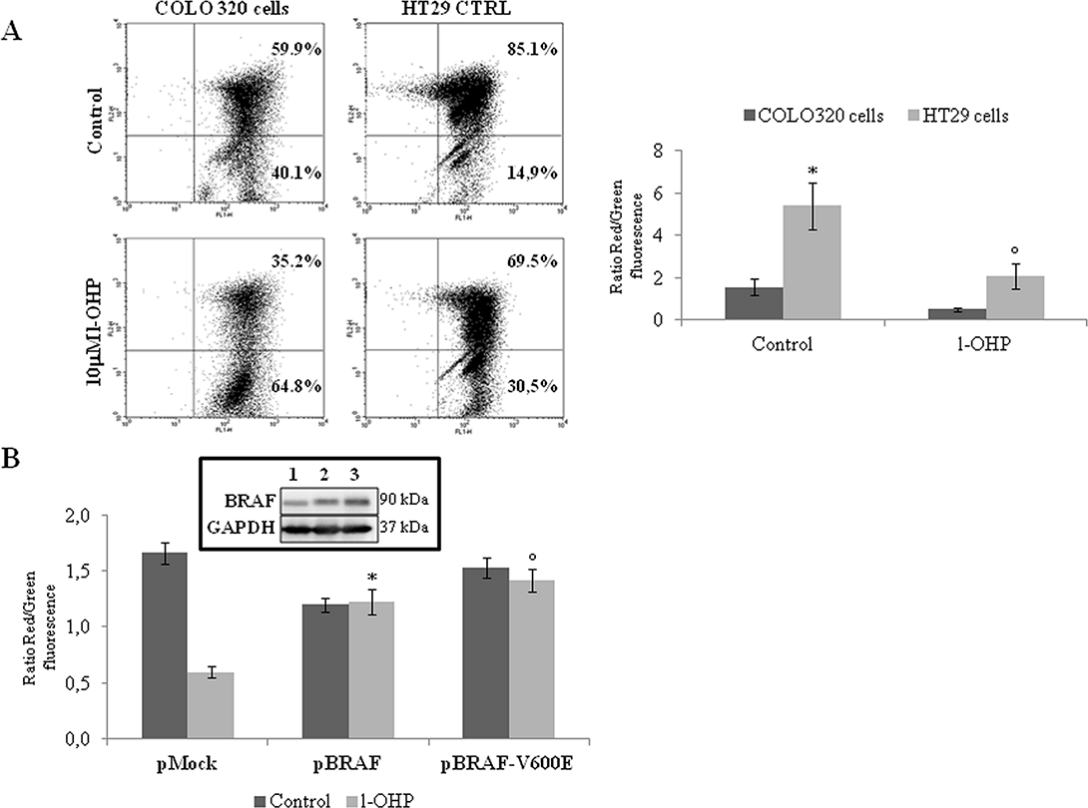
BRAF antiapoptotic activity involves the modulation of mitochondrial apoptotic pathway **A.** Dot plot of fluorescence shift from red to green in COLO320 and HT29 cells treated with 10 μM l-OHP for 24 h. The histogram reports the average result of 3 independent experiments, expressed as ratios between red and green fluorescence. Statistical significance respect to COLO320 cells: *p = 0.005; °*p* = 0.01. **B.** Ratios between red and green fluorescence in COLO320 cells transfected with BRAF wild type or BRAF-V600E constructs and exposed to 10 μM l-OHP for 24 h. Statistical significance respect to pMock cells treated with l-OHP: *p = 0.001; °*p* = 0.0002. **Insert**: Immunoblot analysis of BRAF expression in COLO320 cells transfected with pMock (1), pBRAF wild type (2) or pBRAF-V600E (3) constructs.

Since it is well known that human BRAF-addicted CRCs are characterized by reduced responsiveness to chemotherapeutics [9–11], we evaluated the drug sensitivity of BRAF-mutated compared to BRAF-wild type (wt) human CRC cell lines. Indeed, BRAF-V600E HT29 and COLO205 cells showed poor sensitivity to oxaliplatin (l-OHP) and irinotecan (IRI) compared to BRAF-wt COLO320 cells (Figure 1A), as well as transfection of either BRAF-wt or the BRAF-V600E mutant in COLO320 cells resulted in reduced sensitivity to l-OHP and IRI (Figure 1B). Consistently, BRAF silencing increased l-OHP-induced apoptotic cell death in BRAF-V600E HT29 cells to an extent similar to TRAP1 silencing (Figure 1C). In order to explore further whether BRAF antiapoptotic response involves inhibition of the mitochondrial apoptotic pathway, l-OHP-induced mitochondrial depolarization was evaluated in BRAF-wt COLO320 in comparison to BRAF-V600E HT29 cells (Figure 2A), and in COLO320 cells transfected with BRAF-wt or BRAF-V600E constructs (Figure 2B). In actual fact, BRAF-V600E HT29 cells exhibited higher mitochondrial basal polarization and reduced depolarization in response to l-OHP compared to COLO320 cells (Figure 2A), as well as upregulation of BRAF-wt or the BRAF-V600E constructs protected from mitochondrial depolarization in COLO320 cells upon exposure to l-OHP (Figure 2B). These data suggest that BRAF protects from apoptosis by inhibiting the mitochondrial apoptotic pathway.

### The antiapoptotic function of BRAF is TRAP1-dependent

**Figure 3:**
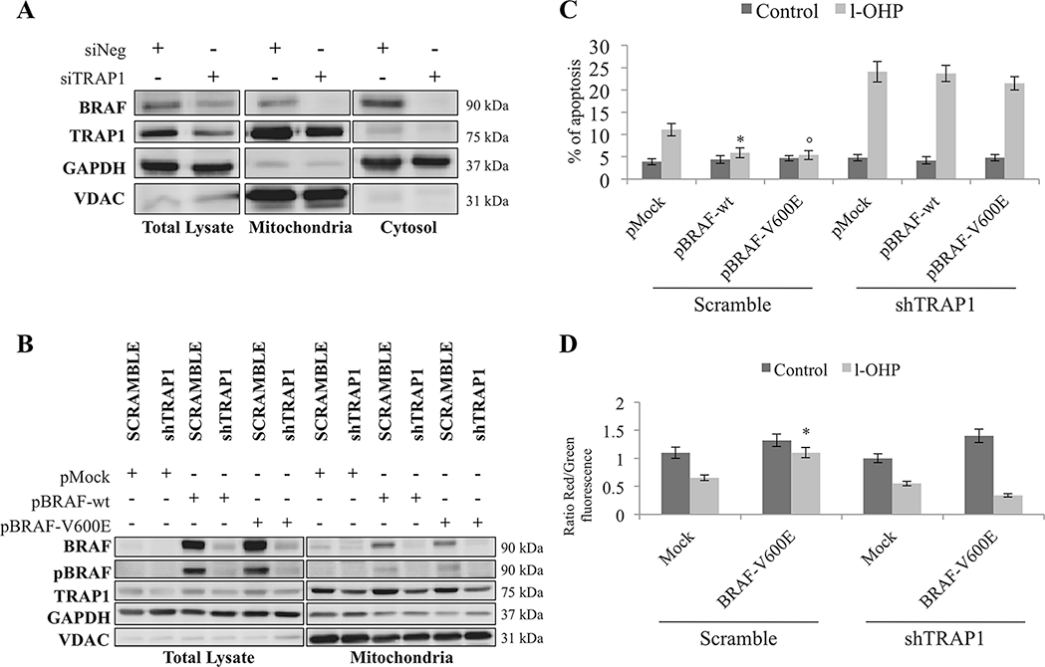
BRAF antiapoptotic activity is TRAP1-dependent **A.** Total lysates and mitochondria and cytosolic fractions were obtained from COLO320 cells transiently silenced for TRAP1 by siRNAs. Equal amounts of proteins were separated by SDS–PAGE and immunoblotted with indicated antibodies. **B.** Total lysates and mitochondria fractions were obtained from scramble and shTRAP1 HCT116 cells transfected with BRAF wild type or BRAF-V600E constructs. Equal amounts of proteins were separated by SDS–PAGE and immunoblotted with indicated antibodies. **C.** Apoptotic levels in scramble and shTRAP1 HCT116 cells transfected with BRAF wild type cDNA or BRAF-V600E mutant and exposed to 10 μM l-OHP for 24 h. Statistical significance respect to pMock cells treated with l-OHP: *p = 0.007; °*p* = 0.005. **D.** Ratios between red and green fluorescence in scramble and shTRAP1 HCT116 cells transfected with the BRAF-V600E mutant and exposed to 10 μM l-OHP for 24 h. Statistical significance respect to pMock cells treated with l-OHP: *p = 0.002.

BRAF subcellular compartmentalization was evaluated in TRAP1-silenced COLO320 cells, since it has been suggested that BRAF signaling alters cell responses to apoptotic stimuli upon traslocation to mitochondria [7] and that TRAP1 regulates BRAF expression/ubiquitination at the translational level [12]. Indeed, TRAP1 silencing resulted in the downregulation of endogenous BRAF in both cytosolic and mitochondrial fractions (Figure 3A). In parallel experiments, cDNAs encoding for BRAF-wt or its V600E mutant were transfected in scramble and shTRAP1 CRC HCT116 cells and cell lysates evaluated for BRAF expression. It is noteworthy that stable TRAP1 interference resulted in lower BRAF mitochondrial basal levels and reduced BRAF upregulation upon transfection of both wild type and V600E constructs (Figure 3B). This evidence is consistent with our previous observation of lower BRAF levels and ERK activation in TRAP1-silenced CRC COLO320 cells transfected with BRAF-wt and BRAF-V600E constructs [12], suggesting that BRAF mitochondrial levels are reduced in a TRAP1-low background.

Considering that mitochondrial TRAP1 is responsible for cytoprotective responses based on its capacity to protect cells from the opening of the MTP [13], we subsequently questioned whether the BRAF antiapoptotic function is TRAP1-dependent. To this aim, drug-induced apoptosis was evaluated in shTRAP1 CRC HCT116 (Figure 3C and Supplementary Figure 1A) and breast carcinoma (BC) MCF7 (Supplementary Figure 1B–1C) cells upon transfection of either BRAF-wt or BRAF-V600E constructs. Indeed, the up-regulation of both BRAF-wt and V600E constructs failed to protect against l-OHP- or paclitaxel-induced apoptosis in shTRAP1 HCT116 (Figure 3C and Supplementary Figure 1A) and MCF7 (Supplementary Figure 1B–1C) cells. Consistently, the upregulation of BRAF-V600E mutant failed to protect from l-OHP-induced mitochondrial depolarization (Figure 3D) in shTRAP1 HCT116 cells. These data suggest that BRAF cytoprotective function requires TRAP1 antiapoptotic activity.

### BRAF interacts with TRAP1 and favors its serine phosphorylation

**Figure 4:**
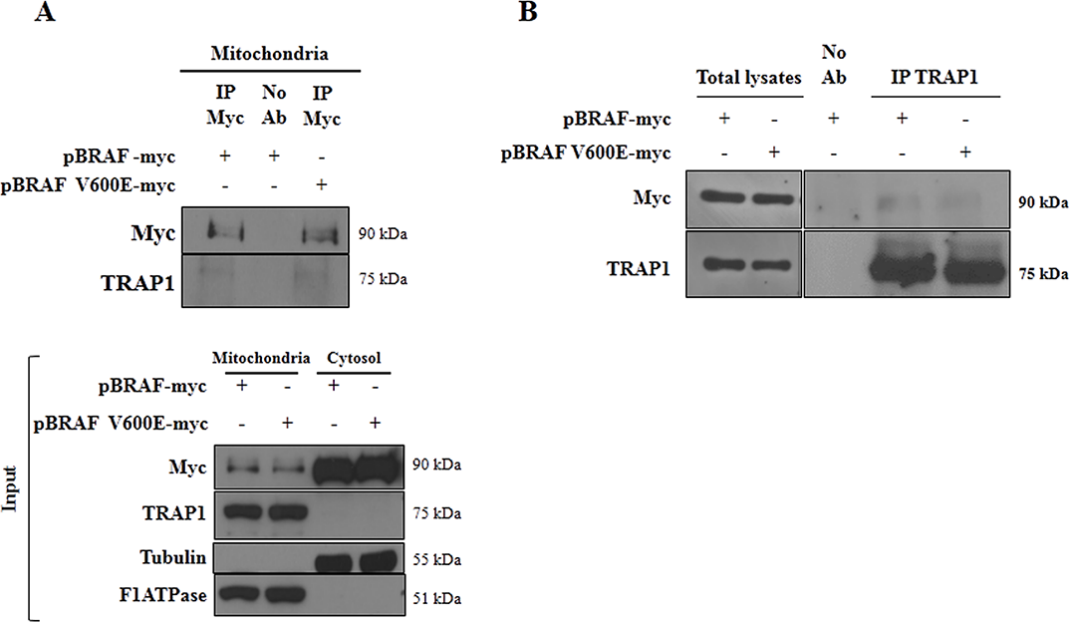
Interaction between TRAP1 and BRAF **A.** Mitochondrial fractions from HCT116 cells transfected with BRAF wild type (BRAF-myc) or BRAF-V600E (BRAF-V600E-myc) constructs were immunoprecipitated with anti-myc antibodies as described in Materials and Methods, separated by SDS-PAGE and immunoblotted with the indicated antibodies. Input: Mitochondrial and cytosolic fractions from HCT116 cells transfected with BRAF wild type (BRAF-myc) or BRAF-V600E (BRAF-V600E-myc) constructs were separated by SDS-PAGE and immunoblotted with the indicated antibodies. **B.** Total lysates from MCF7 cells transfected with BRAF wild type (BRAF-myc) or BRAF-V600E (BRAF-V600E-myc) constructs were immunoprecipitated with anti-TRAP1 antibodies as described in Materials and Methods, separated by SDS-PAGE and immunoblotted with the indicated antibodies. **A–B.** No Ab, total cellular extracts incubated with A/G plus agarose beads without antibody; IP, immunoprecipitation with the corresponding antibodies.

The interaction between BRAF and TRAP1 was explored further: BRAF and TRAP1 co-immunoprecipitation (co-ip) was evaluated in mitochondrial lysates from HCT116 cells co-transfected with TRAP1 and BRAF-wt or its V600E mutant (Figure 4A). Interestingly, the immunoblot analysis showed a band of 75kDa immunoreactive with TRAP1 antibody upon immunoprecipitation of both BRAF-wt and BRAF-V600E (Figure 4A). In reciprocal experiments, TRAP1 and BRAF co-ip was confirmed in total lysates of BC MCF7 cells co-transfected with TRAP1 and BRAF upon TRAP1 immunoprecipitation (Figure 4B).

**Figure 5:**
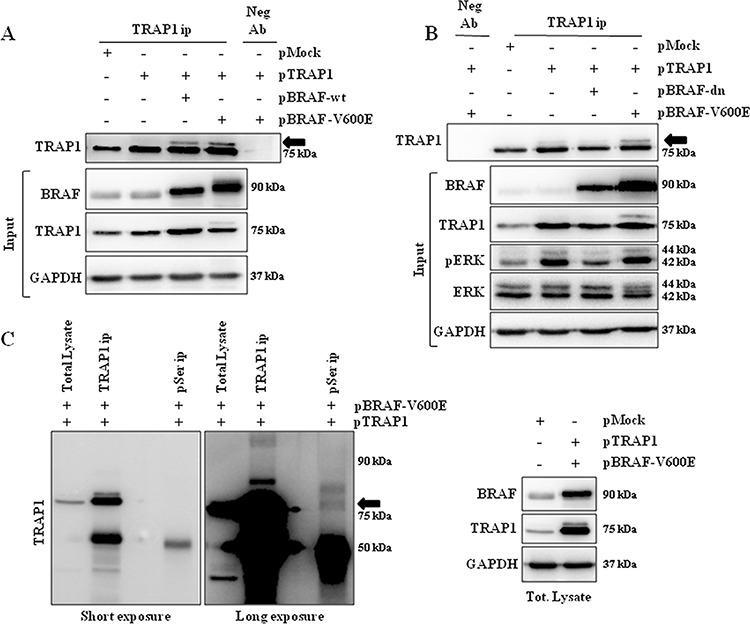
TRAP1 is serine-phosphorylated upon activation of BRAF signaling **A.** Total lysates from CaCo2 cells transfected with TRAP1 or co-transfected with TRAP1 and BRAF-wt or BRAF-V600E constructs were immunoprecipitated with anti-TRAP1 antibodies as described in Materials and Methods, separated by SDS-PAGE and immunoblotted with the indicated antibodies. The arrow indicates the upper band of TRAP1 doublet. Input: Total lysates obtained from cells described in A were separated by SDS-PAGE and immunoblotted with the indicated antibodies. **B.** Total lysates from CaCo2 cells transfected with TRAP1 or co-transfected with TRAP1 and BRAF-V600E or with TRAP1 and BRAF dominant negative (BRAF-dn) constructs were immunoprecipitated with anti-TRAP1 antibodies as described in Materials and Methods, separated by SDS-PAGE and immunoblotted with the indicated antibodies. The arrow indicates the upper band of TRAP1 doublet. Input: Total lysates obtained from cells described in B were separated by SDS-PAGE and immunoblotted with the indicated antibodies. **A–B.** Neg Ab, total cellular extracts incubated with non related antibody; IP, immunoprecipitation with the corresponding antibodies. **C – Right Panel.** Total lysates from HT29 cells transfected with pMock or co-transfected with TRAP1 and BRAF-V600E constructs were separated by SDS-PAGE and immunoblotted with the indicated antibodies. **Left Panel.** Total lysate from HT29 cells co-transfected with TRAP1 and BRAF-V600E constructs was immunoprecipitated with anti-TRAP1 or anti-phosphoserine antibodies as described in Materials and Methods, separated by SDS-PAGE and immunoblotted with the indicated antibodies. The arrow indicates the TRAP1 doublet.

As BRAF is a serine/threonine kinase responsible for the phosphorylation of several intracellular specific substrates [2], we further evaluated whether the activation of BRAF signaling correlates with enhanced TRAP1 serine-phosphorylation. BRAF-wt CaCo2 cells were transfected with TRAP1 alone or co-transfected with TRAP1 and BRAF-wt or the V600E mutant and immunoprecipitated with TRAP1 antibody. Interestingly, TRAP1 immunoblot analysis showed a TRAP1-specific additional band, mostly evident in BRAF and TRAP1 co-transfectants (Figure 5A). It is worth noting that the upper band of the TRAP1 doublet is enhanced in cells co-transfected with the BRAF-V600E constitutively active mutant, also detectable in total lysate (Figure 5A, input). In order to evaluate further the role of BRAF signaling in inducing TRAP1 serine phosphorylation, CaCo2 cells were co-transfected with TRAP1 and a mutant of BRAF acting as dominant negative (BRAF-dn) [22] or the BRAF-V600E construct. Interestingly, the BRAF-dn mutant downregulated ERK phosphorylation in total lysates and prevented TRAP1 serine-phosphorylation, as induced by the BRAF-V600E mutant (Figure 5B). In order to determine whether this TRAP1 doublet is serine phosphorylated, TRAP1 and the BRAF-V600E mutant were co-transfected in HT29 cells (Figure 5C, right panel) and total lysates were immunoprecipitated with either anti-TRAP1 or anti-phosphoserine antibodies and resolved by SDS-PAGE in the same gel (Figure 5C, left panel). Interestingly, TRAP1 immunoblot analysis recognized the 75kDa doublet in both immunoprecipitates (Figure 5C, left panel). Taken as a whole, these data suggest that TRAP1 serine phosphorylation is enhanced in a high BRAF background.

### BRAF silencing/inhibition results in reduced TRAP1 antiapoptotic activity

**Figure 6:**
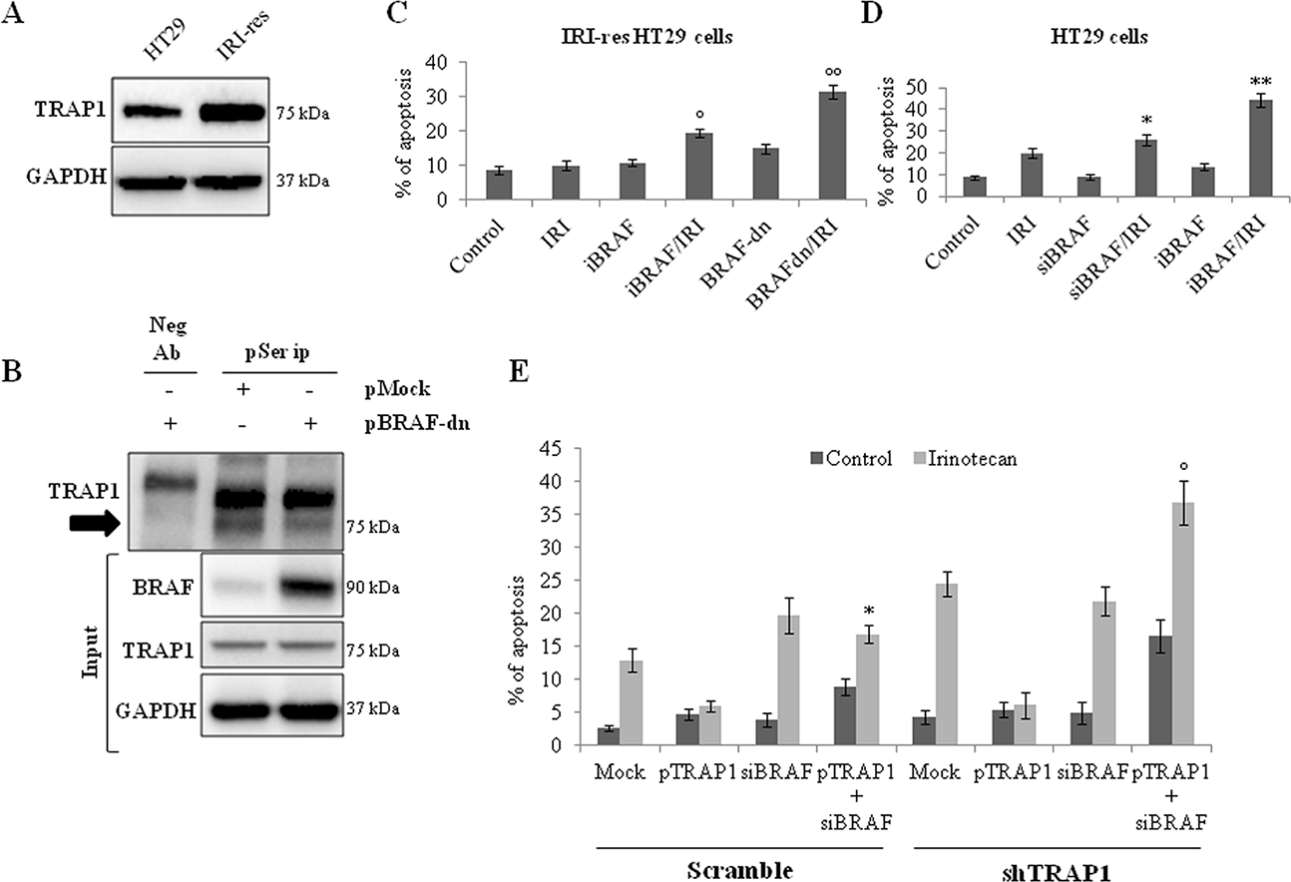
BRAF inhibition prevents TRAP1 serine phosphorylation and re-establish drug-sensitivity in irinotecan-resistant CRC cells **A.** Total lysates from HT29 and irinotecan-resistant (IRI-res) HT29 cells were separated by SDS-PAGE and immunoblotted with the indicated antibodies. **B.** Total lysates from IRI-res HT29 cells transfected with pMock or BRAF dominant negative (BRAF-dn) mutant were immunoprecipitated with anti-phosphoserine antibodies as described in Materials and Methods, separated by SDS-PAGE and immunoblotted with the indicated antibodies. The arrow indicates the phosphorylated form of TRAP1. Neg Ab, total cellular extracts incubated with non related antibody; IP, immunoprecipitation with the corresponding antibodies. Input: Total lysates obtained from the same experimental conditions were separated by SDS-PAGE and immunoblotted with the indicated antibodies. **C–D.** Irinotecan-induced (10 μM IRI for 24 h) apoptosis in irinotecan-resistant HT29 cells transfected with BRAF dominant negative (BRAF-dn) mutant or pretreated with 10 mM vemurafenib for 15 h **(C)** and in HT29 cells transfected with BRAF siRNA or pretreated with 10mM vemurafenib for 15 h **(D)** Statistical significance respect to cells exposed to IRI single agent: °*p* = 0.02; °°*p* = 0.0001; *p = 0.03; **p = 0.0003. **E.** Apoptotic cell death in scramble and shTRAP1 HCT116 cells co-transfected with TRAP1 cDNA and BRAF siRNA and subsequently cultured in the presence and absence of 10 μM IRI for 24 h. Statistical significance respect to pTRAP1-transfected cells treated with IRI: *p = 0.0003; °*p* = 0.0002.

The relevance of TRAP1 serine phosphorylation for antiapoptotic activity was further evaluated in BRAF-V600E IRI-resistant HT29 cells, a tumor cell line characterized by higher TRAP1 levels compared to its drug-sensitive counterpart (Figure 6A) [14] and, thus, suitable for the study of phosphorylation levels of endogenous TRAP1 and the relevance of TRAP1 serine-phosphorylation for antiapoptotic activity. Of note, the transfection of BRAF-dn mutant resulted in reduced serine-phosphorylation of endogenous TRAP1 in IRI-resistant cells (Figure 6B), and rescued sensitivity of IRI-resistant HT29 cells to the specific anticancer agent to an extent similar to BRAF inhibition by vemurafenib (Figure 6C and Supplementary Figure 2A–2B). In parallel experiments, BRAF inhibition/silencing resulted in higher apoptotic rates in response to irinotecan in drug-sensitive HT29 cells (Figure 6D and Supplementary Figure 2C). Finally, the relevance of BRAF for TRAP1 cytoprotective activity was explored by re-expressing TRAP1 in shTRAP1 HCT116 cells upon transient BRAF silencing (Supplementary Figure 2D). It is worth noting here that TRAP1 upregulation protected scramble and shTRAP1 cells from irinotecan-induced apoptosis, whereas its cytoprotective activity was lost in tumor cell lines silenced for BRAF (Figure 6E). These data suggest that TRAP1 antiapoptotic activity is enhanced in a BRAF-rich background and is likely connected to its serine phosphorylation.

### BRAF-addicted colorectal carcinoma cells are highly sensitive to HSP90 chaperones inhibition

**Figure 7:**
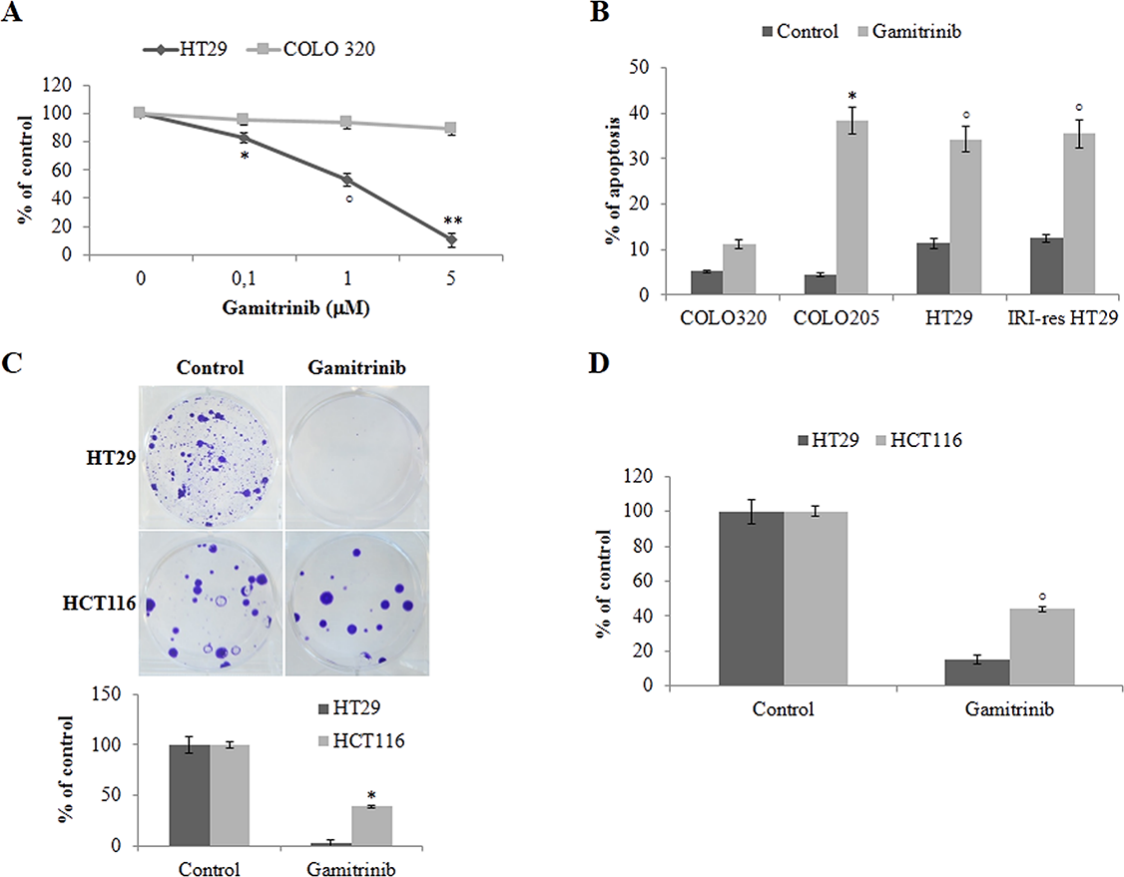
BRAF-mutant colorectal carcinoma cells are highly sensitive to TRAP1 inhibition **A.** Cell viability evaluated by MTT incorporation in BRAF wild type COLO320 and BRAF-V600E HT29 cells. Statistical significance respect to COLO320 cells: *p = 0.01; °*p* = 0.0004; **p < 0.00001. **B.** Apoptotic cell death in BRAF wild type COLO320 and BRAF-V600E COLO205 and HT29 and irinotecan-resistant (IRI-res) HT29 cells exposed to 10 μM gamitrinib for 24 h. Statistical significance respect to COLO320 cells treated with Gamitrinib: *p = 0.0001; °*p* = 0.0002. **C–D.** Foci **(C)** and colony **(D)** formation assay in BRAF wild type HCT116 and BRAF-V600E HT29 cells exposed to 10 μM gamitrinib for 24 h after seeding. The histograms report the average results of 3 independent experiments. Statistical significance respect to gamitrinib-treated HCT116 cells: *p* < 0.00001; °*p* = 0.0001.

Since previous data suggest that BRAF protection from apoptosis is TRAP1-dependent, we questioned whether BRAF-mutated CRC cells are more sensitive to TRAP1 inhibition. To this aim, BRAF-V600E and BRAF-wt CRC cells were exposed to sub-cytotoxic concentrations of the HSP90/TRAP1 dual inhibitor gamitrinib and evaluated for cell viability (Figure 7A), apoptotic cell death (Figure 7B) and colony/foci formation (Figure 7C). Interestingly, HSP90 chaperone inhibition significantly reduced viability of BRAF-V600E HT29 cells compared to BRAF-wt COLO320 cells (Figure 7A) and induced higher levels of apoptosis in BRAF-V600E drug-sensitive and drug-resistant BRAF-mutated cell lines (Figure 7B). Colony- and foci-forming ability was tested in HT29 and HCT116 cells exposed to gamitrinib for 24 h after seeding. Interestingly, gamitrinib inhibited foci (Figure 7C) and colony (Figure 7D) formation in both tumor cell lines, with BRAF-V600E HT29 cells treated with gamitrinib showing a significantly lower colony/foci forming ability compared to BRAF-wt HCT116 cells (Figure 7C–7D). These data suggest that HSP90 chaperones targeting may represent a potential therapeutic strategy in BRAF-addicted CRC cell lines.

## DISCUSSION

Aberrant activation of the RAS/RAF/ERK axis is responsible for favoring several features of human malignancies [2, 3, 5], including uncontrolled cell proliferation, cell survival and metastatic behavior [3, 5]. Furthermore, data overwhelmingly suggest that this pathway is aberrantly activated in human cancer, mostly by mutations of EGFR or RAS and RAF downstream components [5]. Intriguingly, both RAF and ERK translocate to mitochondria where they are involved in cytoprotective functions. Indeed, mitochondrial ERK is responsible for MTP desensitization and resistance to cell death through the modulation of glycogen synthase kinase-3-dependent phosphorylation of the pore regulator cyclophilin D [23]. Furthermore, activated BRAF promotes cell survival by inducing the expression or the phosphorylation of BCL-2 family members [24, 25] and suppresses apoptotic responses against staurosporine and TNFα/cycloheximide in thyroid carcinoma cells [7]. Interestingly, mitochondrial BRAF-V600E inhibits cytochrome c release from mitochondria, favoring resistance to apoptosis. This activity is unaffected by inhibition of ERK activity [7], thus suggesting that mutant BRAF might be responsible by itself for altered responses to apoptotic stimuli within mitochondria. In the clinical perspective, BRAF oncogenic activation confers a worse prognosis to human colorectal [26–29] and thyroid [30] carcinomas and melanomas [31, 32] and is linked to unresponsiveness to traditional and molecular targeted anticancer agents [9–11] and radioiodine [33].

Based on our recent observation that BRAF is a client protein of TRAP1 [12], an HSP90 molecular chaperone upregulated in several human cancers including CRC [13–16], we evaluated the molecular mechanisms responsible for resistance to apoptosis induced by BRAF activation in human CRCs. Our data suggest that i) BRAF antiapoptotic activity involves inhibition of the MTP opening and is TRAP1-dependent, ii) TRAP1 and BRAF interact, with BRAF signaling activation correlated with TRAP1 serine phosphorylation, iii) TRAP1 serine phosphorylation likely favors its antiapoptotic activity and iv) BRAF-addicted CRC cell lines are highly sensitive to both BRAF and TRAP1 targeting. Accordingly, this study verifies the concept that inhibition of TRAP1 chaperoning activity represents a strategy for targeting dependency of BRAF-addicted tumor cells on TRAP1 quality control and antiapoptotic pathway.

These data provide new evidence regarding the reciprocal regulation between TRAP1 chaperoning functions and the BRAF signaling pathway. In previous studies we demonstrated that BRAF synthesis/ubiquitination is regulated at the translational level by TRAP1 in ER [12]. Thus, BRAF expression is enhanced in a high TRAP1 background *in vitro* and in human CRCs. Additionally, TRAP1 silencing/inhibition correlates with lower BRAF synthesis and increase of its ubiquitination, reduced ERK activation, arrest of cell cycle in G0-G1 and G2-M transitions along with wide reprogramming of gene expression with down-regulation of several genes involved in cell cycle progression [12]. Here, consistent with previous observations, we report that BRAF mitochondrial expression and its capacity to inhibit the opening of the MTP and protect from apoptosis are significantly impaired in a low TRAP1 background. Thus, our findings suggest that, in addition to the previously described TRAP1 regulation on BRAF synthesis/ubiquitination in the ER [12], further control exists since TRAP1 represents a downstream effector of BRAF cytoprotective pathway in mitochondria. Indeed, BRAF signaling activation results in induction of TRAP1 serine phosphorylation, which likely enables TRAP1 antiapoptotic function through inhibition of the MTP opening. In such a scenario, the regulation of TRAP1 function by BRAF likely contributes to the enhancement of the apoptotic threshold of cancer cells and induces drug resistance in human BRAF-driven malignancies with TRAP1 upregulation, through the downstream inhibition of the mitochondrial apoptotic pathway. At the same time, TRAP1 overexpression likely represents a mechanism to enhance BRAF synthesis, reduce its ubiquitination and activate its downstream signaling through the ER quality control function [12]. Accordingly, our data suggest dual and reciprocal regulation between the TRAP1 antiapoptotic network and BRAF signaling, likely to be relevant in favoring the apoptosis resistant phenotype shown by human BRAF-mutated malignancies. Still unsolved is the question as to whether TRAP1 is directly phosphorylated by BRAF or by other signaling molecules downstream to BRAF and whether this occurs within or outside mitochondria. The hypothesis that intermediate signaling molecules might mediate TRAP1 serine phosphorylation upon activation of BRAF signaling cannot be ruled out, and represents an issue that merits further investigation.

It is intriguing that the dependency on TRAP1 quality control and survival pathway may represent a mechanism of addiction in BRAF-mutated CRC cells. Consistently with previous data from Altieri's group showing that BRAF-mutated melanoma cells exhibit increased sensitivity to gamitrinib-induced cell death, compared to wild type BRAF melanoma cells [17], CRC cell lines and drug-resistant CRC cells showed high sensitivity to subcytotoxic concentrations of gamitrinib with higher apoptotic rates and impaired colony and foci formation. Furthermore, BRAF inhibition enhanced drug-induced cell death in BRAF-addicted CRC cell lines and the transfection of a BRAF dominant negative mutant prevented TRAP1 serine phosphorylation, as well as re-establishing drug sensitivity in irinotecan-resistant CRC cells, thus reinforcing the concept that the drug-resistant phenotype of this tumor cell model is addicted to the TRAP1/BRAF reciprocal regulatory mechanism. Taken as a whole, these observations support the notion that TRAP1 quality control and antiapoptotic protein network is a potential molecular target for anticancer therapy and that BRAF-addicted tumors are a suitable and attractive tumor cell model to evaluate this novel therapeutic strategy. These data are extremely relevant in the perspective to design new therapeutic strategies and novel combination therapies of different molecular targeted agents in human BRAF-driven CRCs, a subset of colorectal tumors with poor prognosis [26–28] and low response to standard therapies and EGFR monoclonals [9–11]. Indeed, although recently the combination of standard chemotherapy with bevacizumab has been proposed as the best therapeutic option for BRAF-mutated advanced CRCs [34], the prognosis of these patients is still dismal compared to other molecular subtypes of colon cancers [10, 27]. Thus, the development of novel effective therapies represents a clinical need in BRAF-mutant CRCs and, seen in this light, our data provide a strong rationale to design novel specific TRAP1 inhibitors and evaluate BRAF mutational status as a potential biomarker in the selection of tumors suitable for TRAP1 targeting therapy.

## MATERIALS AND METHODS

### Cell cultures, constructs, siRNAs and chemicals

Human CRC HCT116, HT29, COLO320, COLO205 and CaCo2 and BC MCF7 cells were purchased from American Type Culture Collection (ATCC). Cell lines were routinely monitored in our laboratory by microscopic morphology, while cell line authentication was verified before starting this study by STR profiling, according to ATCC product description. MCF7, HCT116, HT29, and CaCo2 cells were cultured in DMEM supplemented with 10% (v/v) fetal bovine serum, 1.5 mM glutamine, and 100 U/ml penicillin and streptomycin, COLO320 and COLO205 cells in RPMI medium 1640 supplemented with 10% (v/v) fetal bovine serum, 0.75 mM glutamine, and 100 U/ml penicillin and streptomycin. TRAP1-stable interfered CRC HCT116 and BC MCF7 cells [19, 35] and drug-resistant CRC cell lines [14, 36] were cultured as previously described.

Full-length TRAP1 construct was obtained as previously described [19, 35], BRAF-wt, BRAF-V600E and BRAFdn constructs kindly provided by Prof. Massimo Santoro (University of Naples Federico II, Naples, Italy) [22]. All constructs were cloned in pcDNA3.1 vector (Invitrogen). Transient transfection of DNA plasmids was performed with Polyfect Transfection reagent (Qiagen), according to manufacturer protocol.

SiRNAs of TRAP1 and BRAF were purchased from Qiagen (Cat. No. SI00115150 for TRAP1, Cat. No. SI00299488 for BRAF). For control experiments, cells were transfected with a similar amount of control siRNA (Qiagen, Cat. No.SI03650318). For knock-down experiments, siRNAs were diluted to a final concentration of 40 nM and transiently transfected by the HiPerFect Transfection Reagent (Qiagen), according to manufacturer protocol.

Gamitrinib was kindly provided by Dr. Altieri (The Wistar Institute, Philadelphia, PA, USA). Unless otherwise specified, reagents were purchased from Sigma-Aldrich.

### Apoptosis assay

Apoptosis was evaluated by citofluorimetric analysis of Annexin-V and 7-amino-actinomycin-D (7-AAD)-positive cells using the fluorescein isothiocyanate (FITC)-Annexin-V/7-AAD kit (Beckman Coulter, Milan, Italy). Stained cells were analyzed using the FACSCalibur™ (Becton Dickinson). Positive staining for Annexin-V as well as double staining for Annexin-V and 7-AAD were interpreted as signs of early and late phases of apoptosis respectively [37].

### Focus and colony forming assays

For focus forming assay, cells were seeded at a density of 200 cells/well in 6-well plates, treated 24 h later with 10 μM gamitrinib for 24 h, and left at confluence for 15 days with medium changes every 3 days. For colony formation assay, 1.25 × 10^4^ cells were suspended in pre-warmed (40°C) 0.7% agarose solution containing 10% (v/v) FBS DMEM, seeded on the top of a bottom layer of a 0.8% agar gel containing 10% (v/v) FBS DMEM, treated with gamitrinib for 24 h and left growing for 15 days as described above. Fifteen days after treatment, the plates were fixed with methanol/acetic acid solution (1:7) and colored with crystal violet. Density of transformation foci/colonies were compared by cell counts and represented as average ± SD.

### Immunoblot analysis

Total cell lysates were obtained by homogenization of cell pellets in a cold lysis buffer (20 mM Tris pH 7.5 containing 300 mM sucrose, 60 mM KCl, 15 mM NaCl, 5% (v/v) glycerol, 2 mM EDTA, 1% (v/v) Triton X-100, 1 mM PMSF, 2 mg/ml aprotinin, 2 mg/ml leupetin and 0.2% (w/v) deoxycholate) for 2 min at 4°C and further sonication for 30 sec on ice. Mitochondria were purified by Qproteome Mitochondria Isolation kit (Qiagen) according to manufacturer protocol. Immunoblot analysis was performed as previously reported [38]. Protein immunoprecipitation was carried out starting from 1 mg of total protein extracts. Lysates were pre-cleared by incubating with protein A/G-Agarose (Santa Cruz Biotechnologies) for 1 h at 4°C and then incubated with gentle shaking for 18 h at 4°C with specific antibodies, after which samples were further incubated for 1 h at 4°C with fresh beads. Beads were collected by centrifugation and washed twice in lysis buffer. The following antibodies from Santa Cruz Biotechnology were used: mouse monoclonal anti-HSP75 (sc-73604), mouse monoclonal anti-cMyc (sc-40), mouse monoclonal anti-BRAF (sc-5284), mouse monoclonal anti-GAPDH (sc-47724), mouse monoclonal anti-Tubulin (sc-8035), mouse monoclonal anti-ATP5B (3D5) (sc-58618). The following antibodies were also used: mouse monoclonal anti-phospho44/42 MAPK (pErk1/2, #9106) and rabbit polyclonal anti-phosphoBRAF (Ser445, #2696) from Cell Signaling Technology, rabbit polyclonal anti-MAPK 1/2 (Erk1/2, #ABS44), rabbit polyclonal anti-VDAC (#AB10527) from Merk Millipore; mouse monoclonal anti-phosphoSerine (#37430) from Qiagen.

### Mitochondrial membrane potential evaluation

Mitochondrial membrane potential was detected by using JC-10 Mitochondrial Membrane Potential Assay Kit – Flow Cytometry (Abcam, ab112133). Cells were seeded into 6-well plates, treated as indicated in the Results, trypsinized, washed with PBS and incubated with JC-10 probe at 37°C for 20 min in the dark. As a control, cells were pre-incubated with 10 μM carbonyl cyanide m-chlorophenylhydrazone (CCCP) at 37°C for 20 min to obtain complete mitochondrial depolarization. Cell fluorescence was measured using the FACSCalibur™ cytometer (Becton Dickinson) and reported as the ratio between red and green fluorescence.

### Statistical analysis

Two-sided paired *T*-test was used to establish statistical differences in apoptosis, ratio of mitochondrial depolarization, and colony/foci formation between BRAF-mutated and BRAF-wt cells, transfected/silenced and non transfected/silenced cells or drug- and vehicle-treated cells. Statistically significant values (*p* < 0.05) are reported in Figure Legends. All experiments were independently performed at least three times.

## SUPPLEMENTARY FIGURES


