# Supplementary material for: Targeting TRAP1 as a downstream effector of BRAF cytoprotective pathway: A novel strategy for human BRAF-driven colorectal carcinoma
Source: Oncotarget. 2015 Jun 13;6(26):22298–309. doi: 10.18632/oncotarget.4263 (PMC4673164; doi:10.18632/oncotarget.4263)
Supplement: Supplementary file 1 [file oncotarget-06-22298-s001.pdf]

SUPPLEMENTARY FIGURES

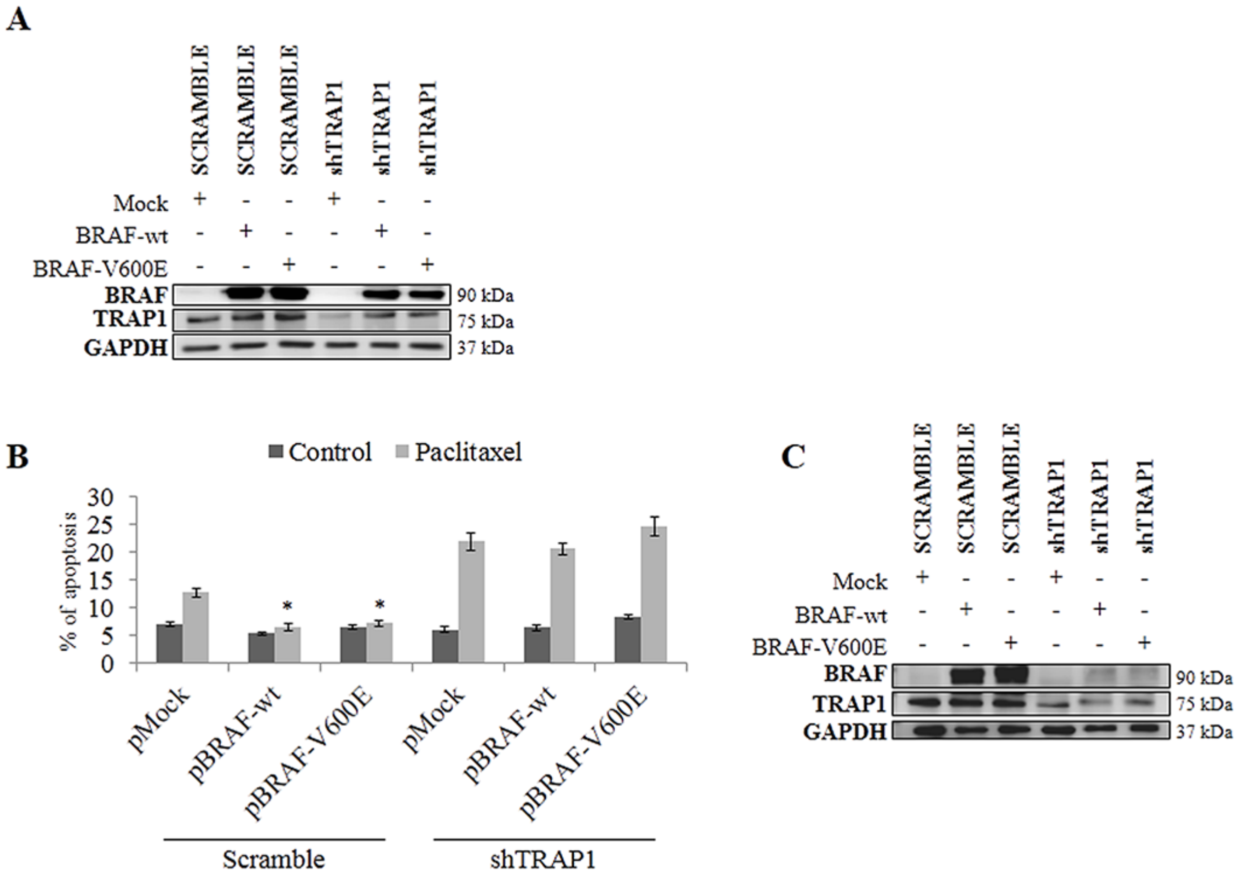

Supplementary Figure S1: A. Total lysates were obtained from scramble and shTRAP1 HCT116 cells transfected with BRAF wild type cDNA or BRAF-V600E mutant. Equal amounts of proteins were separated by SDS–PAGE and immunoblotted with indicated antibodies. Samples represent expressions controls of apoptotic data reported in Figure 3C. **B.** Apoptotic levels in scramble and shTRAP1 MCF7 cells transfected with BRAF wild type cDNA or BRAF-V600E mutant and exposed to 10 µM paclitaxel for 24 h. Statistical significance respect to pMock cells treated with paclitaxel: *p = 0.0008. **C.** Total lysates were obtained from scramble and shTRAP1 MCF7 cells transfected with BRAF wild type cDNA or BRAF-V600E mutant. Equal amounts of proteins were separated by SDS–PAGE and immunoblotted with indicated antibodies. Samples represent expressions controls of apoptotic data reported in Panel B.

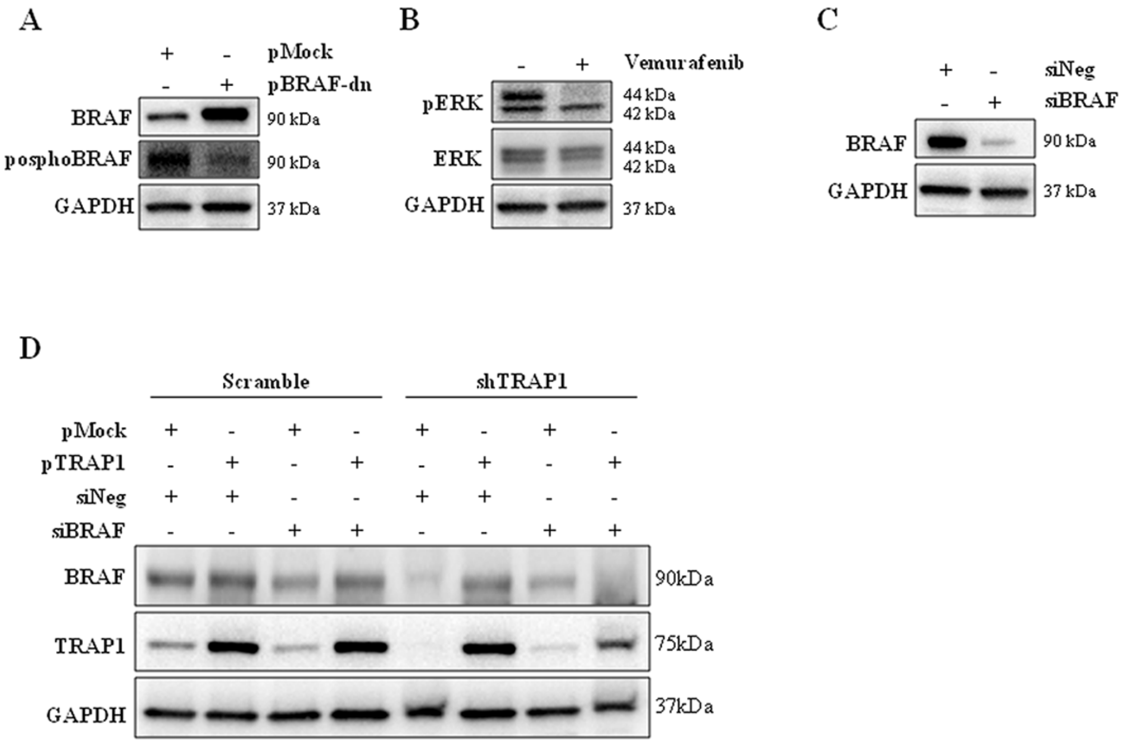

Supplementary Figure S2: A–B. Total lysates from irinotecan-resistant HT29 cells transfected with BRAF dominant negative (BRAF-dn) mutant (A) or treated with 10 mM vemurafenib for 15 h (B) were separated by SDS–PAGE and immunoblotted with indicated antibodies. Samples represent expressions controls of apoptotic data reported in Figure 6C. **C**. Total lysates from HT29 cells transfected with Negative or BRAF siRNAs were separated by SDS–PAGE and immunoblotted with indicated antibodies. Samples represent expressions controls of apoptotic data reported in Figure 6D. **D**. Total lysates from scramble and shTRAP1 HCT116 cells transfected with pTRAP1 and/or BRAF siRNA were separated by SDS–PAGE and immunoblotted with indicated antibodies. Samples represent expressions controls of apoptotic data reported in Figure 6E.
